# Supplementary material for: Overlapping Streptococcus pyogenes and Streptococcus dysgalactiae subspecies equisimilis household transmission and mobile genetic element exchange
Source: Nat Commun. 2024 Apr 24;15:3477. doi: 10.1038/s41467-024-47816-1 (PMC11043366; doi:10.1038/s41467-024-47816-1)
Supplement: Supplementary file 3 — Description of Additional Supplementary Files [file 41467_2024_47816_MOESM3_ESM.pdf]

<u>Description of Additional Supplementary Files</u>

Supplementary Data 1. a) List of 294 *Streptococcus dysgalactiae* subspecies *equisimilis* (SDSE) isolates with whole genome sequencing data included in the study with associated clinical and genomic metadata. **b)** Virulence and antimicrobial resistance gene presence and absence across the 294 SDSE isolates. **c)** Presence of sil locus and bacteriocin biosynthetic clusters in 315 *Streptococcus pyogenes* isolates. Bacteriocin biosynthetic clusters predicted by antiSMASH v7.1.0. Full *S. pyogenes* molecular typing data available at *Lacey* et al.[1]
