# Supplementary material for: Overlapping Streptococcus pyogenes and Streptococcus dysgalactiae subspecies equisimilis household transmission and mobile genetic element exchange
Source: Nat Commun. 2024 Apr 24;15:3477. doi: 10.1038/s41467-024-47816-1 (PMC11043366; doi:10.1038/s41467-024-47816-1)
Supplement: Supplementary file 5 — Reporting Summary [file 41467_2024_47816_MOESM5_ESM.pdf]

nature portfolio

Corresponding author(s): Prof Steven Tong

Last updated by author(s): Mar 27, 2024

Reporting Summary

Nature Portfolio wishes to improve the reproducibility of the work that we publish. This form provides structure for consistency and transparency in reporting. For further information on Nature Portfolio policies, see our Editorial Policies and the Editorial Policy Checklist.

Statistics

For all statistical analyses, confirm that the following items are present in the figure legend, table legend, main text, or Methods section.

n/a	Confirmed	
☐	☒	The exact sample size (*n*) for each experimental group/condition, given as a discrete number and unit of measurement
☐	☒	A statement on whether measurements were taken from distinct samples or whether the same sample was measured repeatedly
☐	☒	The statistical test(s) used AND whether they are one- or two-sided<br>*Only common tests should be described solely by name; describe more complex techniques in the Methods section.*
☐	☒	A description of all covariates tested
☐	☒	A description of any assumptions or corrections, such as tests of normality and adjustment for multiple comparisons
☐	☒	A full description of the statistical parameters including central tendency (e.g. means) or other basic estimates (e.g. regression coefficient) AND variation (e.g. standard deviation) or associated estimates of uncertainty (e.g. confidence intervals)
☐	☒	For null hypothesis testing, the test statistic (e.g. *F*, *t*, *r*) with confidence intervals, effect sizes, degrees of freedom and *P* value noted<br>*Give P values as exact values whenever suitable.*
☒	☐	For Bayesian analysis, information on the choice of priors and Markov chain Monte Carlo settings
☒	☐	For hierarchical and complex designs, identification of the appropriate level for tests and full reporting of outcomes
☒	☐	Estimates of effect sizes (e.g. Cohen's *d*, Pearson's *r*), indicating how they were calculated

Our web collection on statistics for biologists contains articles on many of the points above.

Software and code

Policy information about availability of computer code

Data collection	No software was used for data collection
Data analysis	All custom code and scripts used to analyse the data are publicly available at https://github.com/OuliXie/Strep_MGE_pipeline and https://github.com/OuliXie/SDSE_transmission.<br>Software packages used in the analysis include Kraken2 v2.1.2, emmtyper v0.2.0, MLST v2.22.0, PopPUNK v.2.60, Panaroo v1.2.10, Corekaburra v0.0.5, IQ-tree v2.0.6, Snippy v4.6.0, phangorn v2.10.0, SKA v1.0, CD-HIT v4.8.1, minimap2 v2.24, survival v3.4.0, survminer v0.4.9, igraph v1.3.5, ggraph v2.1.0, scatterpie v0.1.8, and ape v5.7.

For manuscripts utilizing custom algorithms or software that are central to the research but not yet described in published literature, software must be made available to editors and reviewers. We strongly encourage code deposition in a community repository (e.g. GitHub). See the Nature Portfolio guidelines for submitting code & software for further information.

Data

Policy information about availability of data

All manuscripts must include a data availability statement. This statement should provide the following information, where applicable:

- Accession codes, unique identifiers, or web links for publicly available datasets
- A description of any restrictions on data availability
- For clinical datasets or third party data, please ensure that the statement adheres to our policy

The sequence data generated in this study have been deposited in the European Nucleotide Archive under BioProject identifier PRJEB35476 (https://www.ebi.ac.uk/ena/browser/view/PRJEB35476). The full de-identified clinical data are available under restricted access for ethical and privacy purposes; reasonable requests for access can be discussed by contacting the corresponding author by email. The processed epidemiological data used to generate these analyses are available in Supplementary Data 1a. S. pyogenes sequences have previously been published17 and are available under BioProjects PRJNA879913 (https://www.ncbi.nlm.nih.gov/bioproject/?term=PRJNA879913) and PRJEB2232 (https://www.ebi.ac.uk/ena/browser/view/PRJEB2232). Source data are provided with this paper. The authors confirm all supporting data have been provided within the article or in supplementary data files.

Research involving human participants, their data, or biological material

Policy information about studies with human participants or human data. See also policy information about sex, gender (identity/presentation), and sexual orientation and race, ethnicity and racism.

Reporting on sex and gender	No reporting on sex or gender was performed
Reporting on race, ethnicity, or other socially relevant groupings	Although the race or ethnicity of individual participants was not reported, the two communities included in this study were described as remote Australian First Nations/Aboriginal communities in the Northern Territory of Australia. No community identifiers are reported. This description was required to provide context to the transmission dynamics described and the significance of findings in the setting of the current and historically high burden of streptococcal disease in remote Aboriginal communities in the Northern Territory of Australia.
Population characteristics	Skin and throat swabs were collected between August 2003 to June 2005 in two remote Aboriginal communities in the Northern Territory of Australia. Both adults and children were included in the study as well as routine use of intramuscular benzathine penicillin for rheumatic heart disease prophylaxis. Only 11 participants were receiving benzathine penicillin for rheumatic heart disease prophylaxis across the two communities. A median of 28 people (range 6–57) were enrolled per household over the study period. Age of participants was previously described by McDonald et al. (DOI: 10.3201/eid1311.061258).
Recruitment	Isolates were collected from a previously reported prospective surveillance study (McDonald et al. DOI: 10.1086/506938) in three remote Aboriginal communities in remote Northern Territory, Australia, which were visited approximately monthly over a two-year period from August 2003 to June 2005. Due to waning community support and logistical difficulties in community 2, it was replaced with another community in June 2004 (community 3). Only communities 1 and 3 were included in this study. At each visit, researchers collected throat swabs regardless of symptoms from consenting participants and examined for skin sores both purulent and dry, which were also swabbed. Households were chosen based on a history of residents with rheumatic heart disease or acute rheumatic fever. Due to high population mobility, individuals were identified as part of households for analyses, including family groups residing in one or two adjacent houses. As each individual was observed at a median of 3 visits (range 1–19, intermittently sampled), analyses at an individual level could not be performed.
Ethics oversight	The current study received ethics approval from the Human Research Ethics Committee of the Northern Territory Department of Health and Menzies School of Health Research (approval 2015-2516).

Note that full information on the approval of the study protocol must also be provided in the manuscript.

Field-specific reporting

Please select the one below that is the best fit for your research. If you are not sure, read the appropriate sections before making your selection.

☒ Life sciences ☐ Behavioural & social sciences ☐ Ecological, evolutionary & environmental sciences

For a reference copy of the document with all sections, see nature.com/documents/nr-reporting-summary-flat.pdf

Life sciences study design

All studies must disclose on these points even when the disclosure is negative.

Sample size	No formal sample size calculations were performed. However, the sample size was chosen to be sufficiently large to infer transmission chains by inclusion of all available Streptococcus dysgalactiae subsp. equisimilis (294/330, 89%) and Streptococcus pyogenes (315/327, 96%) isolates from the original surveillance study conducted by McDonald et al. These isolates were obtained from 4,547 person-consultations during 486 household-visits including 1,087 individuals (547 from community 1 and 540 from community 3).
Data exclusions	A community with low recruitment (community 2) was excluded as transmission networks were unlikely to be inferred with low sample

Data exclusions	numbers. Sequences failing quality control as described in the methods were excluded.
Replication	Seeds were used for permutation tests and transmission network visualisation to ensure reproducibility. Custom code to classify mobile genetic elements is modified from a previous publication by Xie et al. (DOI: 10.1101/2023.08.10.552873) and is publicly available at https://github.com/OuliXie/Strep_MGE_pipeline. Code to infer transmission networks and permutations tests are available at https://github.com/OuliXie/SDSE_transmission.
Randomization	No randomisation was performed. Participants were recruited from households with a known history of acute rheumatic fever or rheumatic heart disease. Throat swabs were taken regardless of symptoms and skin swabs were taken from skin sores (both wet and dry). Isolates were allocated to their respective species (Streptococcus dysgalactiae subsp. equisimilis or S. pyogenes) based on kraken2 and MLST classification.
Blinding	No blinding was performed in this study. Blinding was not required as the results are quantitative and did not require subjective judgment or interpretation. Prophylactic benzathine penicillin was prescribed by the participant's physician based on a history of acute rheumatic fever/ rheumatic heart disease and was not determined by this study.

Reporting for specific materials, systems and methods

We require information from authors about some types of materials, experimental systems and methods used in many studies. Here, indicate whether each material, system or method listed is relevant to your study. If you are not sure if a list item applies to your research, read the appropriate section before selecting a response.

Materials & experimental systems

n/a	Involved in the study
☒	☐ Antibodies
☒	☐ Eukaryotic cell lines
☒	☐ Palaeontology and archaeology
☒	☐ Animals and other organisms
☒	☐ Clinical data
☒	☐ Dual use research of concern
☒	☐ Plants

Methods

n/a	Involved in the study
☒	☐ ChIP-seq
☒	☐ Flow cytometry
☒	☐ MRI-based neuroimaging

Plants

Seed stocks	*Report on the source of all seed stocks or other plant material used. If applicable, state the seed stock centre and catalogue number. If plant specimens were collected from the field, describe the collection location, date and sampling procedures.*
Novel plant genotypes	*Describe the methods by which all novel plant genotypes were produced. This includes those generated by transgenic approaches, gene editing, chemical/radiation-based mutagenesis and hybridization. For transgenic lines, describe the transformation method, the number of independent lines analyzed and the generation upon which experiments were performed. For gene-edited lines, describe the editor used, the endogenous sequence targeted for editing, the targeting guide RNA sequence (if applicable) and how the editor was applied.*
Authentication	*Describe any authentication procedures for each seed stock used or novel genotype generated. Describe any experiments used to assess the effect of a mutation and, where applicable, how potential secondary effects (e.g. second site T-DNA insertions, mosiacism, off-target gene editing) were examined.*
